# Efficient tri-metallic oxides NiCo_2_O_4_/CuO for the oxygen evolution reaction†

**DOI:** 10.1039/c9ra09351f

**Published:** 2019-12-20

**Authors:** Abdul Qayoom Mugheri, Aneela Tahira, Umair Aftab, Adeel Liaquat Bhatti, Nusrat Naeem Memon, Jamil-ur-Rehman Memon, Muhammad Ishaque Abro, Aqeel Ahmed Shah, Magnus Willander, Ahmed Ali Hullio, Zafar Hussain Ibupoto

**Affiliations:** Dr M. A. Kazi Institute of Chemistry, University of Sindh Jamshoro 76080 Sindh Pakistan zaffar.ibhupoto@usindh.edu.pk; Department of Science and Technology, Campus Norrkoping, Linkoping University SE-60174 Norrkoping Sweden; Mehran University of Engineering and Technology 7680 Jamshoro Sindh Pakistan; Institute of Physics, University of Sindh Jamshoro 76080 Sindh Pakistan; NED University of Engineering Science and Technology Karachi Sindh Pakistan

## Abstract

In this study, a simple approach was used to produce nonprecious, earth abundant, stable and environmentally friendly NiCo_2_O_4_/CuO composites for the oxygen evolution reaction (OER) in alkaline media. The nanocomposites were prepared by a low temperature aqueous chemical growth method. The morphology of the nanostructures was changed from nanowires to porous structures with the addition of CuO. The NiCo_2_O_4_/CuO composite was loaded onto a glassy carbon electrode by the drop casting method. The addition of CuO into NiCo_2_O_4_ led to reduction in the onset potential of the OER. Among the composites, 0.5 grams of CuO anchored with NiCo_2_O_4_ (sample 2) demonstrated a low onset potential of 1.46 V *vs.* a reversible hydrogen electrode (RHE). A current density of 10 mA cm^−2^ was achieved at an over-potential of 230 mV and sample 2 was found to be durable for 35 hours in alkaline media. Electrochemical impedance spectroscopy (EIS) indicated a small charge transfer resistance of 77.46 ohms for sample 2, which further strengthened the OER polarization curves and indicates the favorable OER kinetics. All of the obtained results could encourage the application of sample 2 in water splitting batteries and other energy related applications.

## Abstract 2

In this study, a simple approach was used to produce nonprecious, earth abundant, stable and environmentally friendly NiCo_2_O_4_/CuO composites for the oxygen evolution reaction (OER) in alkaline media.

## Graphical abstract

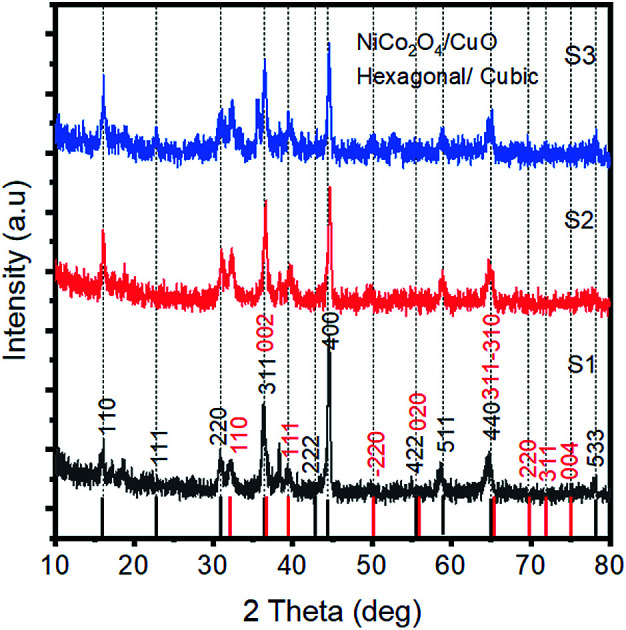

## 1. Introduction

The high consumption of fossil fuels is increasing the energy crisis and creating environment-related problems. This has led to the realization of alternative energy conversion and storage devices, including water catalysis, fuel cells and metal–air batteries.^1–5^ Water catalysis has received significant attention in recent years as it can produce hydrogen and oxygen gases.^6^ Hydrogen generation *via* solar or wind energies and its utilization result in zero carbon emissions into the atmosphere. However, hydrogen is not naturally in excess on earth, and the industrial production of hydrogen needs a high amount of energy. Beside this, solar water dissociation for the generation of hydrogen only uses water and sunlight, and thus it is considered as a unique technology for hydrogen production. However, large over-potential required for the oxygen evolution reaction (OER) has impaired its practical application for water dissociation.^7^ The greatest challenge for the OER is to develop a cost-effective methodology, which would mean that it can be consequently exploited for commercial applications. To date, noble metals such as Ir and Ru have been found to be the most efficient catalysts for the OER.^8–10^ However, their widespread use is restricted due to their high cost and rare abundance. Hence, it is a very challenging task to design nonprecious, highly efficient and durable OER electrocatalysts. For this purpose, a wide range of OER catalysts have been reported in the recent past.^11–14^

Among the non-noble electrocatalysts, oxides of Mn, Fe, Co, Ni and Cu have been produced and extensively investigated for OER activity.^15^ In particular, cobalt-based oxides are highly promising catalysts due to their rich occurrence, facile synthesis and high stability.^16^ The doping strategy into Co_3_O_4_ has been shown to enhance the OER activity compared to pristine Co_3_O_4_.^16^ Furthermore, the nonprecious bimetallic oxide NiCo_2_O_4_ has shown promising properties for the OER, due to favorable properties such as high electronic and ionic conductivities, low cost, stability *etc.*^17,18^ In the crystalline structure of NiCo_2_O_4_, the Ni occupies octahedral positions and cobalt fills both octahedral and tetrahedral positions. It has been found that the redox chemistry of NiCo_2_O_4_ is highly attractive due to the mutual contributions from nickel and cobalt. In recent investigations, it has been shown that NiCo_2_O_4_ exhibits better electrochemical activity and electronic conductivities compared to the single NiO and Co_3_O_4_ counterparts.^19^ A wide range of morphologies of NiCo_2_O_4_ have been prepared and their OER performance evaluated. Zhou *et al.* prepared nanosheets of NiCo_2_O_4_ and used them for the OER, in which a current density of 10 mA cm^−2^ was obtained at the consumption of 1.63 V *versus* a reversible hydrogen electrode (RHE) in 1.0 M KOH solution.^20^ Furthermore, the deposition of NiCo_2_O_4_ onto carbon nanotubes increased the OER activity and resulted in higher OER activity than that found for the pristine NiCo_2_O_4_.^21^ Zhu *et al.* showed that an Fe dopant enhanced the electrocatalytic activity of NiCo_2_O_4_ in 1.0 M KOH conditions and the pristine NiCo_2_O_4_ needs 1.69 V *vs.* RHE to produce a current density of 10 mA cm^−2^.^18^ Xia *et al.* deposited Au–NiCo_2_O_4_ on a graphene-like sheet and observed excellent OER activity^22^ and NiCo_2_O_4_ nanosponges demonstrated superior OER performance.^23^ The importance of NiCo_2_O_4_ can be seen from this literature, and it confirms the unique OER activity of NiCo_2_O_4_, which is attributed to its excellent electrical conductivity, earth-abundant nature, low cost and significant stability in basic solutions. Moreover, the dual participation of nickel and cobalt leads to the rich redox chemistry of NiCo_2_O_4_, making it an emerging and potential material for the OER.^24,25^ Beside this, extensive research has been carried out on copper-based materials for water oxidation applications because of their rich occurrence, inexpensive nature and high redox chemistry.^26^ These studies have indicated that copper-based materials are active for water oxidation, but they exhibit a slightly large onset overpotential in the range of 320–450 mV.^26^ There is a scientific gap for more investigations in order to lower the onset potential of copper-based materials for water oxidation and to uplift the redox properties of NiCo_2_O_4_. Also, motivated by a recent study from our group,^27^ in which we used NiO as a supporting material for the growth of Co_3_O_4_ and found bifunctional water splitting activity in alkaline media, herein, we provide an efficient OER electrocatalyst based on a NiCo_2_O_4_/CuO composite. In this work, we propose that CuO has a poor conductivity, and thus its OER activity is found at higher potential; however, when it is combined with NiCo_2_O_4_, it exhibits superior activity for the oxygen evolution reaction. The synergy of more active sites from CuO and its rich redox chemistry when coupled with a highly electrically conducting material such as NiCo_2_O_4_, facilitates efficient OER activity in alkaline media.

Herein, we used a simple strategy to produce a range of nonprecious catalysts using a wet chemical method. The coupling of NiCo_2_O_4_ with CuO resulted in superior OER activity compared to pristine NiCo_2_O_4_ in alkaline media. A low onset potential of 1.46 V *versus* RHE is found for the composite sample and a favorable Tafel slope is estimated. Also, the composite catalyst is highly durable for 35 hours and EIS has shown low charge transfer resistance and high double layer capacitance.

## 2. Experimental section

Nickel nitrate hexahydrate, copper acetate monohydrate, cobalt nitrate hexahydrate, potassium hydroxide, hexamethylenetetramine, urea, ethanol, 5% Nafion and alumina powder were received from Sigma-Aldrich, Karachi, Pakistan. They were used without further treatment.

The CuO nanostructures were grown by a low temperature aqueous chemical growth method. A solution of 0.1 M copper acetate monohydrate and 0.1 M hexamethylenetetramine was prepared in 100 mL of distilled water. The precursor solution was covered with an aluminum sheet and kept in an electric oven at 95 °C for 5 hours. The copper hydroxide nanostructures were collected by common filter paper and further annealed at 400 °C in air for 4 hours in order to achieve CuO phase. Then, the CuO nanostructures were functionalized with NiCo_2_O_4_*via* a wet chemical method. For the functionalization of NiCo_2_O_4_, 0.1 M cobalt nitrate hexahydrate and 0.1 M urea were used and an impurity of 0.03 M nickel nitrate hexahydrate was also added into 0.3 grams, 0.5 grams and 0.7 grams of CuO in separate beakers containing 100 mL of distilled water, and they were labeled as sample 1 (S1), 2 (S2) and 3 (S3), respectively. Afterwards, the beakers were tightly sealed with an aluminum sheet and left in an electric oven at 95 °C for 5 hours. After the completion of the growth time, the beakers were cooled at room temperature and the nanostructured product was collected on filter paper. Then, the obtained product was annealed at 400 °C in air for 5 hours in order to get trimetallic composite oxide. The structure and shape related features were studied by scanning electron microscopy (SEM) at an accelerating voltage of 3.0 kV and powder X-ray diffraction (XRD) patterns were measured with a Bruker D8-Advance diffractometer using Cu Kα radiation. Energy dispersive spectroscopy (EDS) was used to identify the elements in the tri-metallic oxides.

### 2.1. Electrochemical measurements

Cyclic voltammetry (CV) and linear sweep voltammetry (LSV) were used to perform electrochemical characterization and the instrument was a Versa Potentiostat, Netherlands, in a three electrode configuration using platinum wire as a counter electrode, a silver–silver chloride electrode as a reference electrode and a polished glassy carbon electrode (GCE) as a working electrode, modified with 10 μL of different catalysts using the drop casting method. The polishing of the glassy carbon electrode was performed with alumina powder and a silica sheet as the rubbing surface. A 1.0 M KOH electrolytic solution was used to carry out the electrochemical experiments. The catalyst ink was made by dispersing 10 mg of each catalyst in 2.5 mL of distilled water and 0.5 mL of 5% Nafion solution. A well dispersed catalyst ink was obtained *via* sonication for 20 minutes. Before the LSV experiments, CV was performed 5–10 times at the scan rate of 5 mV s^−1^ in 1.0 M KOH in order to ensure the stability of the material on the GCE. LSV was used to monitor the OER activity at a scan rate of 1 mV s^−1^ in 1.0 M KOH. All the potentials are reported against a reversible hydrogen electrode (RHE) throughout the manuscript. Electrochemical impedance spectroscopy (EIS) was performed at the onset potential of 1.46 V *vs.* RHE and the sweeping frequency range from 100 kHz to 1 Hz at an amplitude of 5 mV. Chronopotentiometry was used to evaluate the stability of the catalyst for 35 hours in 1.0 M KOH solution. For the confirmation of the repeatability of the OER activity, all of the experiments were done 3–6 times. Z-view software was used to simulate the EIS results and the fitted results are presented here.

## 3. Results and discussion

### 3.1. The crystallography and morphology studies of various nanostructured materials

**Fig. 1:**
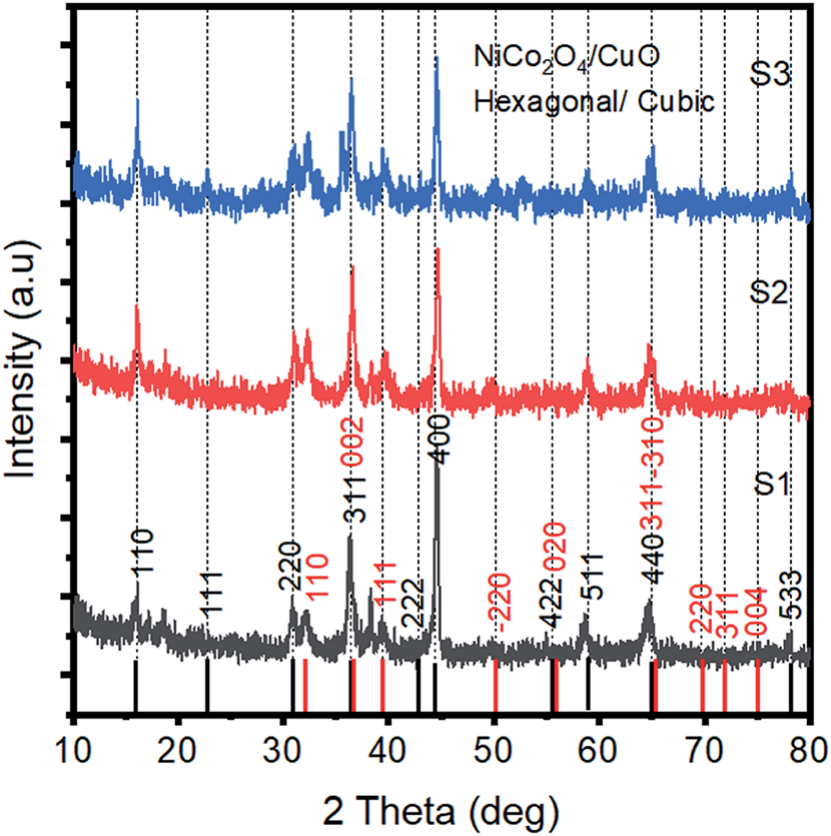
XRD diffraction patterns of the NiCo_2_O_4_/CuO composites S1, S2 and S3.

**Fig. 2:**
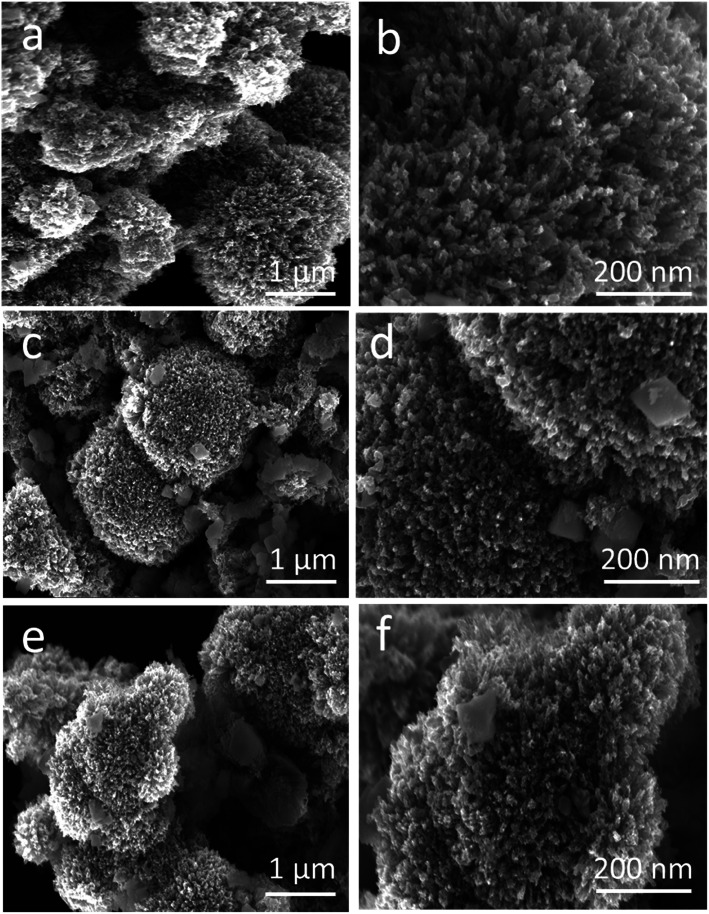
Low and high resolution SEM images of different NiCo_2_O_4_/CuO composites: (a and b) S1, (c and d) S2, and (e and f) S3.

**Fig. 3:**
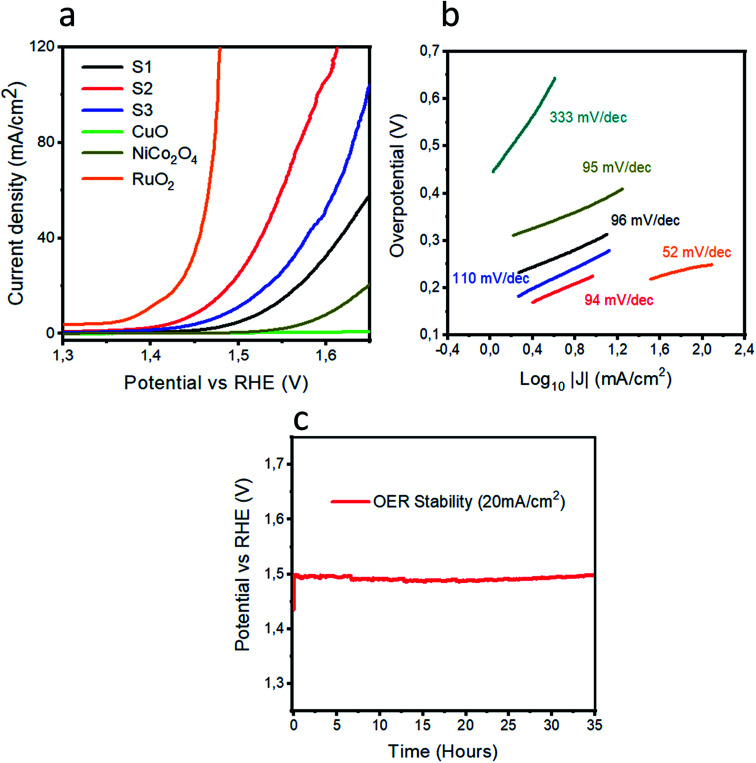
(a) LSV polarization curves of RuO_2_, pristine NiCo_2_O_4_, CuO, S1, S2, and S3 at the scan rate of 1 mV s^−1^ in 1.0 M KOH. (b) Tafel plots of the various materials. (c) The chronopotentiometry experiment showing the durability of S2 for 35 hours at a current density of 20 mA cm^−2^.

Powder XRD was used to explore the existence of different phases within the composite materials, as shown in Fig. 1. The diffraction patterns are correlated to the spinal phase of NiCo_2_O_4_ and well matched to JCPDS card no. 20-0781. Also, some of the CuO reflections are also detected by XRD as shown in Fig. 1 and they are matched to JCPDS card no. 48-1548, and CuO exhibits a monoclinic phase. The XRD study reveals that we have a tri-metallic composite consisting of NiCo_2_O_4_/CuO. However, a weak signal at 18 deg is found, which corresponds to the Co_3_O_4_ phase.^27^ The microstructure and shape orientation were studied for samples 1, 2 and 3 by SEM, as shown in Fig. 2a–f. We found that the addition of CuO caused an alternation in the morphology from nanowires to a particle structure with porous features. The porous structure provided more active sites for the contact of electrolyte, and as a result superior OER activity is observed. Different amounts of CuO showed no significant effect on the change of morphology, so we see approximately similar structures with successive addition of different contents of CuO. The size of the composite materials is between 200–500 nm, as shown in Fig. 2. The pristine CuO and NiCo_2_O_4_ have a petal and nanowire-like morphology, respectively, as depicted in S1A and B. Elemental mapping was carried out on the composite samples S1, S2 and S3, and a uniform distribution of Co, Ni, Cu and O within the samples was found, as shown in S2. The EDS spectra are also shown in S2 for samples S1, S2 and S3 and the presence of Co, Ni, and O was predominant. The OER performance of the as obtained samples was investigated in a 1.0 M KOH solution (pH = 14) *via* a three electrode configuration as shown in Fig. 3. From Fig. 3, RuO_2_ displays an onset potential of 1.42 V *vs.* RHE, whereas the NiCo_2_O_4/_CuO composite has a lower onset potential of 1.46 V *vs.* RHE than the pristine NiCo_2_O_4_ and CuO, and they exhibit superior OER activity. The performance of sample 2 is better than that of sample 1 and 3 in terms of current density and onset potential. The pristine NiCo_2_O_4_ shows a higher onset potential of 1.61 V *vs.* RHE, which is higher than that of sample 2, and this further indicates that the addition of CuO resulting in superior OER activity by providing more active sites and conductivity features from NiCo_2_O_4_ contribute equally towards the excellent OER activity, as shown in Fig. 3. Importantly, the over-potential of sample 2 is only 230 mV to achieve a 10 mA cm^−2^ current density, which is lower than that of the pristine NiCo_2_O_4_ (380 mV). The over-potential of sample 2 at 10 mA cm^−2^ is lower or comparable to those of already reported Co-based OER catalysts^28–52^Fig. 3b shows the Tafel plots obtained from the linear region of the LSV polarization curves and the Tafel plots are in good agreement with the Tafel equation. As seen in Fig. 3b, the Tafel slope of sample 2 is 94 mV dec^−1^, comparable to that of pristine NiCo_2_O_4_ (95 mV dec^−1^), suggesting that the rate determining step is governed by the Volmer mechanism; however, the addition of CuO has lowered the onset potential and over-potential for the OER as demonstrated by sample 2. In a previous study,^53^ oxygen vacancies contributed significantly to the superior OER. These vacancies cause a distortion in the structure of catalyst, which can facilitate the adsorption of H_2_O onto the surface of the materials and speed up the OER process.^54–57^ A high density of oxygen vacancies results in a low Tafel slope value.^47^ The findings show that the Tafel slope of sample 2 is slightly lower than that of pristine NiCo_2_O_4_, which is in good agreement with the LSV polarization curves.

It is a very challenging task to describe the OER mechanism; however, the most widely-accepted mechanism of the OER in alkaline media on transition metal oxides is a multi-proton-coupled four electron transfer reaction, as formulated below:

a M* + OH^−^ → e^−^ + M*OH

b M*OH + OH^−^ → e^−^ + H_2_O + M*O

c M*O + OH^−^ → e^−^ + M*OOH

d M*OOH + OH^−^ → H_2_O + e^−^ + M*OO

e M*OO → O_2_ + M*

where M* shows the availability of active sites in the electrode.^58–61^

From the Tafel slope values, we can take a guideline to illustrate the rate determining step. In our case, the most governing rate-determining steps for the OER in basic conditions are OH^−^ adsorption (a), O–H bond splitting (b) and O_2_ desorption (e), which are assigned to the theoretical Tafel slopes values of 120, 30, and 20 mV dec^−1^ respectively.^62^ The Tafel values are found to be between 30 and 120 mV dec^−1^ for pristine NiCo_2_O_4_, RuO_2_ and the composite samples, revealing that the rate-determining steps are completely mixed and they involve OH^−^ adsorption (a) and O–H bond splitting (b).^63^ After the exploration of the OER activity, it is very vital to investigate the durability for the realization of practical applications of OER electrocatalysts. Chronopotentiometry was used to confirm the durability of sample 2 for a time period of 35 hours, as shown in Fig. 3c. In the durability experiment, sample 2 retains a potential of 20 mA cm^−2^ for the OER and this further gives a deeper insight into the long-term use of sample 2 in practical water splitting battery applications. The performance of sample 2 in terms of over-potential is better or comparable to that of many of the recently designed electrocatalysts, as shown in Table S4.†^69–76^

**Fig. 4:**
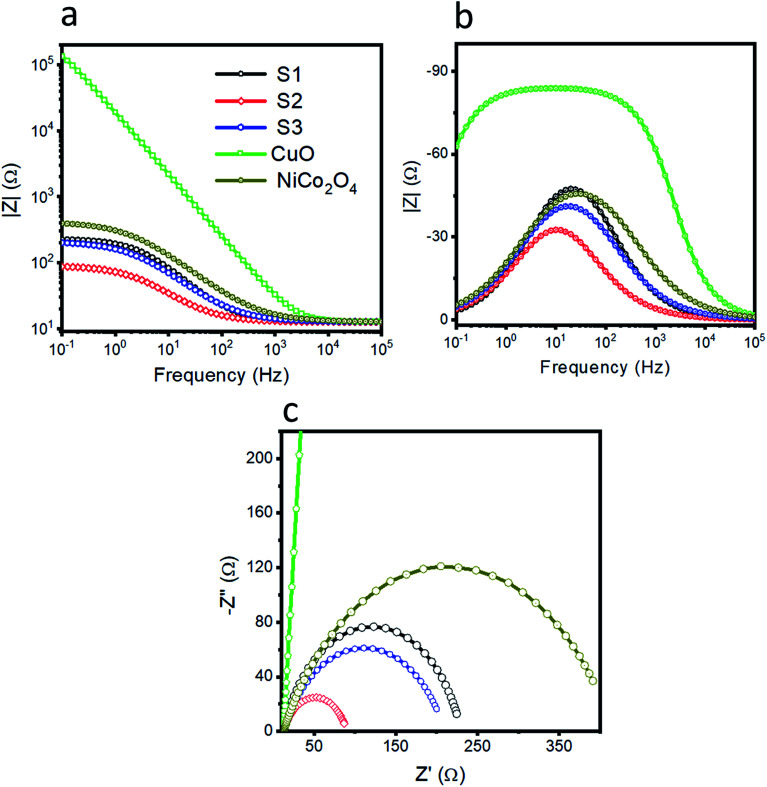
Electrochemical impedance spectroscopy (EIS) was performed at the onset potential of 1.46 V *vs.* RHE and a sweeping frequency range from 100 kHz to 1 Hz at an amplitude of 5 mV. (a) EIS Nyquist spectra for pristine NiCo_2_O_4_, CuO, S1, S2, and S3, and (b and c) their corresponding Bode plots.

The charge transfer resistance between the electrode and electrolyte gives a deeper understanding of the electrochemical reaction to be performed. Thus, electrochemical impedance spectroscopy was used to obtain insightful information about the charge transfer rate of various catalysts, as shown in Fig. 4. The Nyquist plots are shown in Fig. 4a at the onset potential of 1.46 V *vs.* RHE. The Bode plots are also obtained from the same impedance data as represented in Fig. 4b and c. The raw data were fitted through the equivalent circuit model as shown in S3. Different circuit elements were used, such as *R*_s_, related to the solution resistance, which is nearly the same for the different materials. The arc of the curve indicates the magnitude of the charge transfer resistance (*R*_ct_). A small charge transfer resistance suggests a faster charge transfer between the electrode and electrolyte. The simulated charge transfer resistance values for pristine NiCo_2_O_4_, CuO and samples 1, 2 and 3 are 403.4, 259590, 217.6, 77.46, and 197.3 ohms, respectively, as given in Table 1. The low value of the charge transfer resistance for sample 2 reveals swift OER kinetics. The single peak in the Bode plot is attributed to the relaxation, which is correlated to the charge transfer phenomenon. The information that we get from the Bode plots indicates the gain and phase angles of different catalysts with variation in the frequency. Furthermore, the optimum oscillation frequency of the impedance semicircle of sample 2 is low in value compared to the other presented materials, which indicates that sample 2 has a higher electron recombination lifetime (*τ*_*n*_ = 1/2π*f*_max_).^64–68^ The EIS study demonstrates that sample 2 is associated with a small charge transfer resistance and a higher double layer capacitance of 755.33 μF, which strongly supports the obtained LSV OER polarization curves, as shown in Fig. 3a.

**Table 1:** The simulated values of the equivalent circuit elements

Sample	*R*_s_	*R*_ct_	*C*_dl_
Sample 1	12.5	217.6	224.96
Sample 2	12.58	77.46	755.33
Sample 3	12.67	197.3	323.86
NiCo_2_O_4_	12.6	403.4	173.18
CuO	12.7	259590	9.81

## 4. Conclusions

In summary, we present a facile approach for the development of an active NiCo_2_O_4_/CuO composite for the oxygen evolution reaction by a low temperature aqueous chemical method. The coupling of CuO with NiCo_2_O_4_ has shown variation in morphology from nanowires to porous structures. The porous structure of the composite materials, especially sample 2, has several advantageous features, such as high activity, faster OER kinetics, small over-potential of 230 mV, a small charge transfer resistance of 77.46 ohms and excellent durability for 35 hours. The superior performance comes from the synergy of the rich active sites of CuO and the high conductivity of NiCo_2_O_4_, which facilitates the OER activity. Based on the obtained results, it is safe to say that sample 2 can be regarded as a potential and promising material for an extended range of environment and energy-related applications.

## Conflicts of interest

Authors declare no conflict of interest.

## Supplementary Material

RA-009-C9RA09351F-s001
