# Supplementary material for: Efficient tri-metallic oxides NiCo2O4/CuO for the oxygen evolution reaction
Source: RSC Adv. 2019 Dec 20;9(72):42387–94. doi: 10.1039/c9ra09351f (PMC9076628; doi:10.1039/c9ra09351f)
Supplement: RA-009-C9RA09351F-s001 [file RA-009-C9RA09351F-s001.pdf]

Electronic Supplementary Material (ESI) for RSC Advances.
This journal is © The Royal Society of Chemistry 2019

Supplementary data

Efficient tri-metallic oxides for oxygen evolution reaction

Abdul Qayoom Mugheri[a], Aneela Tahira[b], Umair Aftab[c], Adeel Liaquat Bhatti[d], Nusrat Naeem Memon[a],Jameel-ur-Rahman Memona, Muhammad Ishaque Abro[c], Aqeel Ahmed Shah[e], Magnus Willander[b], Ahmed Ali Hullio[a], Zafar Hussain Ibupoto[*a]

[a] Dr. M.A Kazi Institute of Chemistry University of Sindh Jamshoro, 76080, Sindh Pakistan

[b]Department of Science and Technology, Campus Norrkoping, Linkoping University, SE-60174 Norrkoping, Sweden

[c]Mehran University of Engineering and Technology, 7680 Jamshoro, Sindh Pakistan

[d]Institute of Physics University of Sindh Jamshoro, 76080, Sindh Pakistan

[e]NED University of Engineering Science and Technology, Karachi, Sindh Pakistan

Corresponding author: Zafar Hussian Ibupoto, PhD

Email address: zaffar.ibhupoto@usindh.edu.pk

S1:(A) pristine CuO, (B) pristine $NiCo_2O_4$

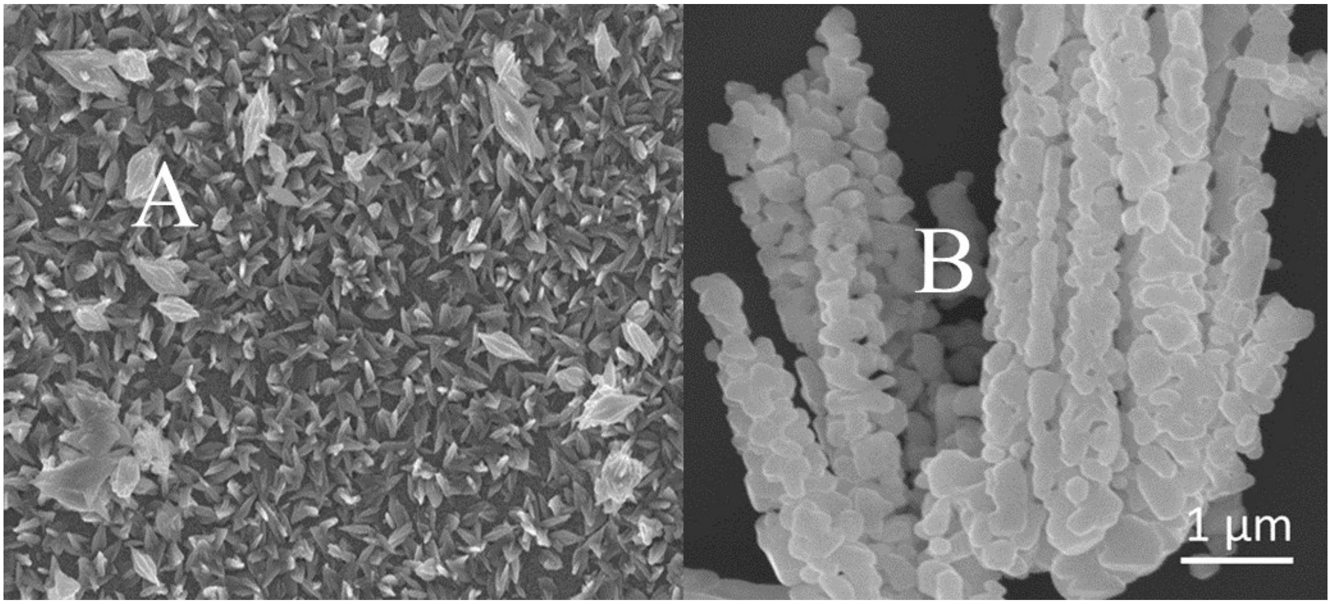

S2:

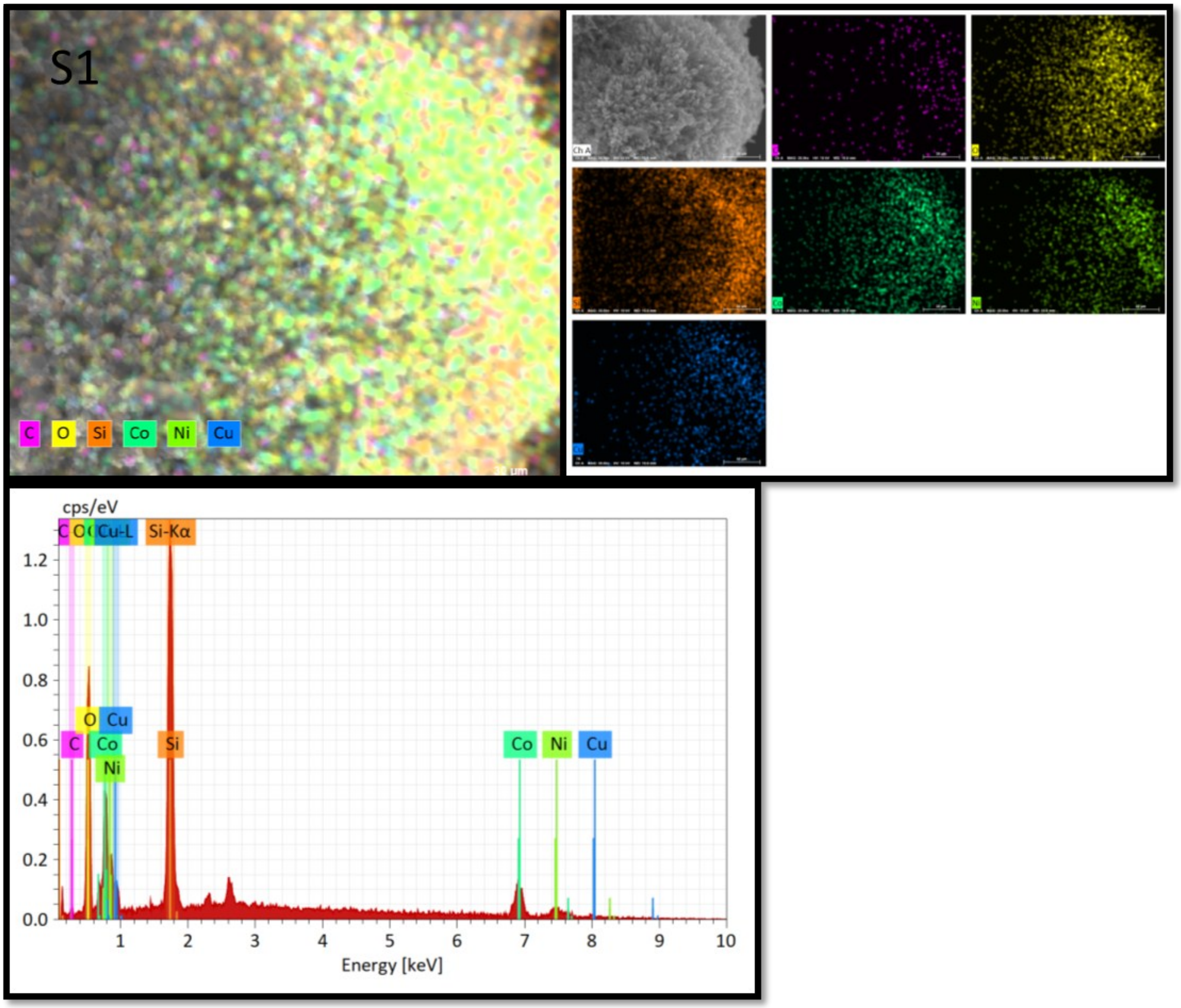
S1
C
O
Si
Co
Ni
Cu
cps/eV
C
O
Cu-L
Si-Kα
1.2
1.0
0.8
0.6
0.4
0.2
0.0
O
Cu
C
Co
Ni
Si
Co
Ni
Cu
1
2
3
4
5
6
7
8
9
10
Energy [keV]

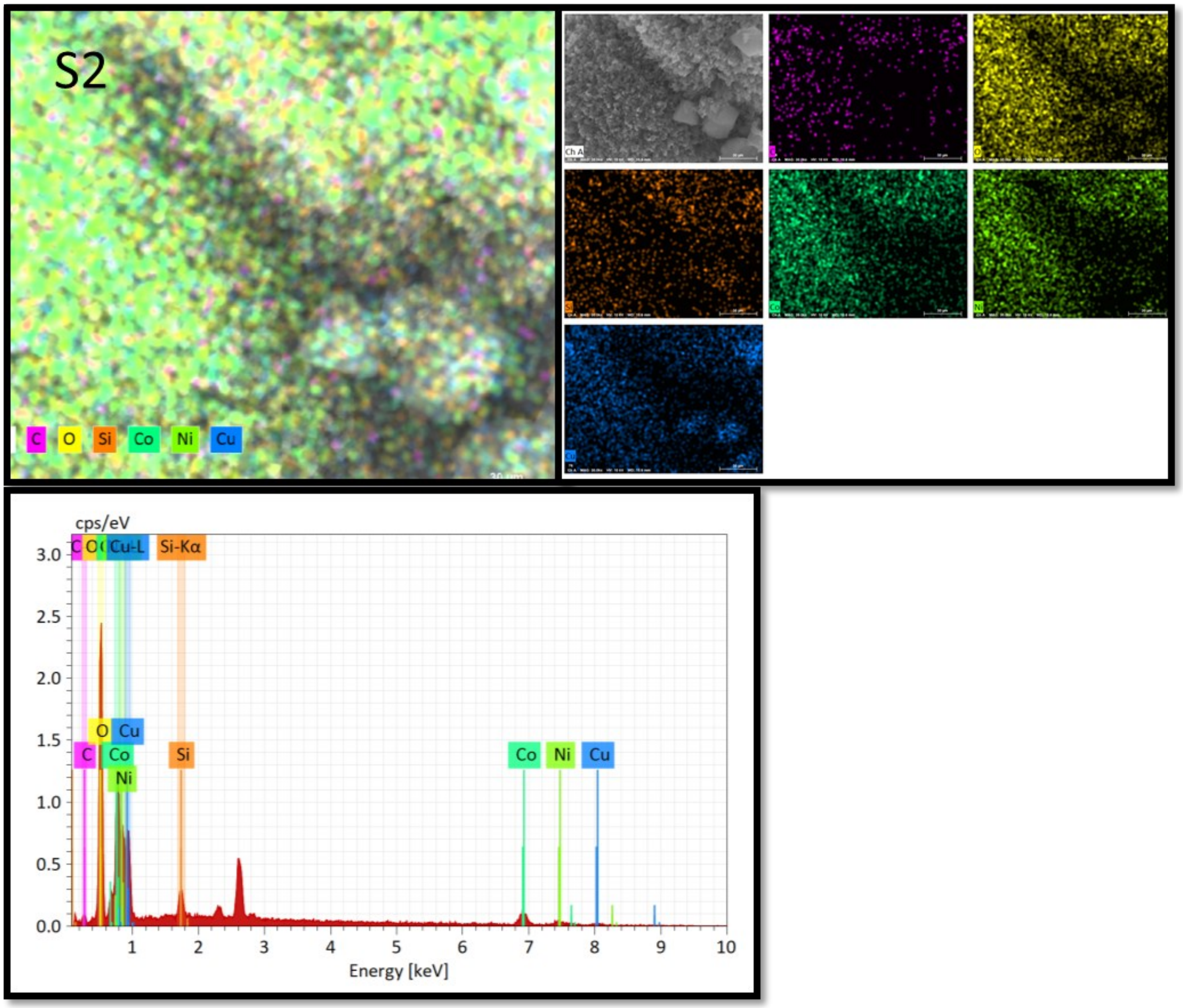
S2
C
O
Si
Co
Ni
Cu
cps/eV
Cu-L
Si-Kα
3.0
2.5
2.0
1.5
1.0
0.5
0.0
1 2 3 4 5 6 7 8 9 10
Energy [keV]

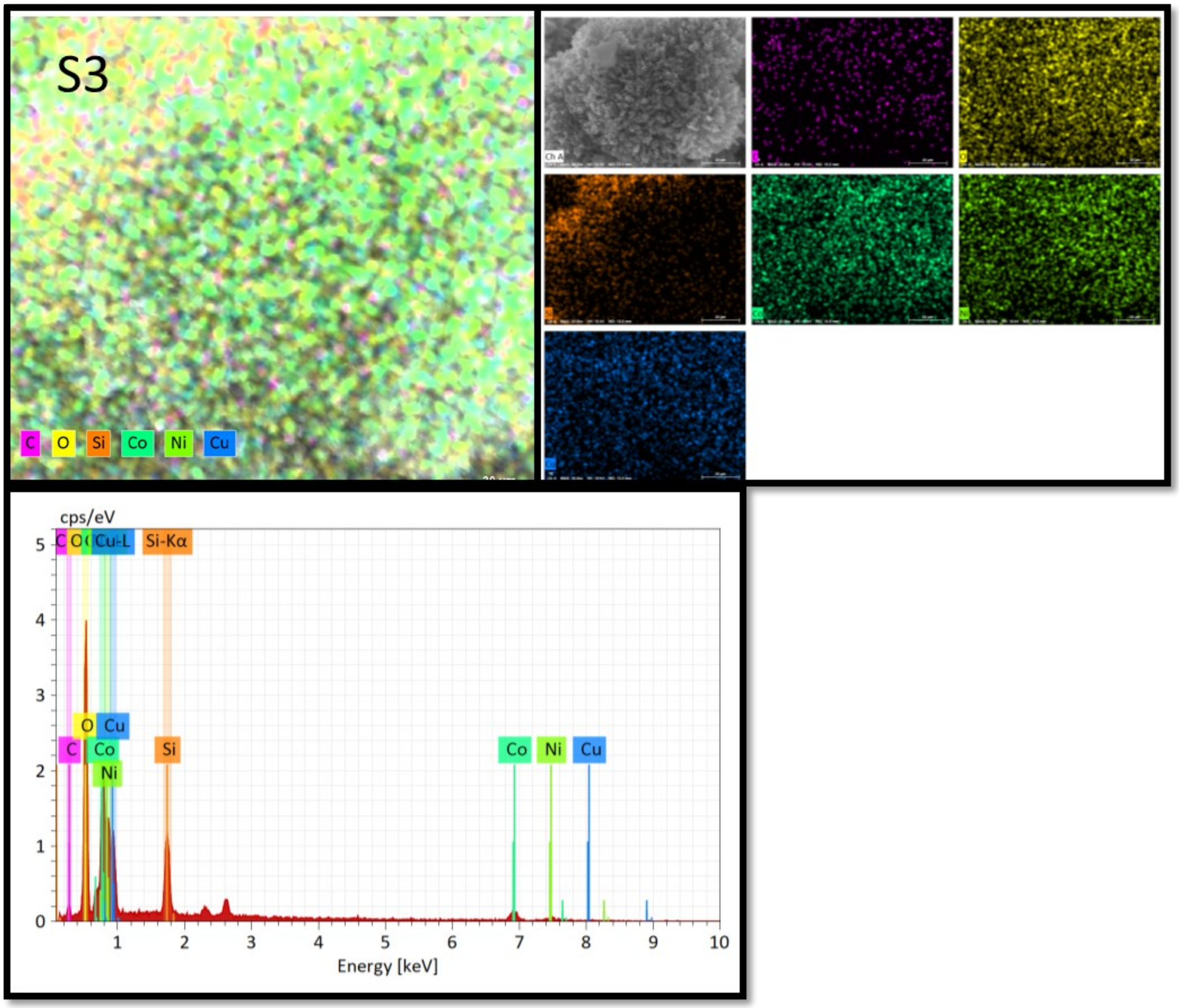

S3: shows the equivalent circuit model for the impedance analysis

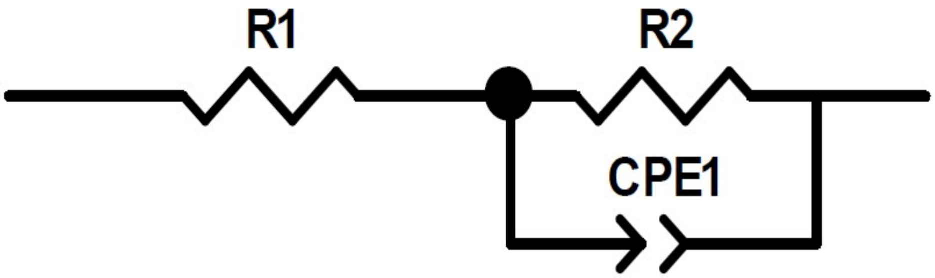

Table S1: Comparison of OER performance-based oxide electrocatalysts in alkaline media with recently reported works.

Catalyst	Tafel slope ($mVdec^{-1}$)	Overpotential () @ 10 mA/cm^2	References.
CoCo LDH	59	393	1
CoOx@CN	N/A	~385	2
Co-P films	47	345	3
$MnCo_2Ox$	84	>410	4
Co_3O_4/N-rmGO	67	310	5
NiCoOx	N/A	420	6
N-G-CoO	71	340	7
$NixCo_3$-xO	59~64	~370	8
$NiCo_2O_4$/CuO	94	230	Present work

References

1. F. Song and X. Hu J. Nature Communications 2014, 5, 4477.

2. H. Jin, J. Wang, D. Su, Z. Wei, Z. Pang and Y. Wang J. Am. Chem. Soc. 2015, 137, 7, 2688-2694.

3. N. Jiang D. B. You and M. Sheng, J. Angewandte Chemie. 2015, 18, 6251-6254

4. F. Song and X. Hu J. Am. Chem. Soc., 2014, 1364716481-16484.

5. Y. Liang, Y. Li, H. Wang, J. Zhou, J. Wang and T. Regier J. Nature Materials, 2011, 10, 780–786.

6. C. C. L. McCrory, S. Jung, J. Peters J. Am. Chem. Soc., 2013, 26-28.

7. X. Cui, P. Ren, D. Deng, J. Deng and X. Bao, J. Energy Environ. Sci., 2016, 9, 123-129.

8. Y. Li, P. Hasin Y. Li and P. Hasin J. advanced materials., 2010, 4, 1926-1929.
